# To the Medical Public

**Published:** 1845-11

**Authors:** M. C. Johnson

**Affiliations:** Chairman of the Board of Trustees, Transylvania University; Lexington, Kentucky


### MEDICAL INTELLIGENCE.

Lexington, Kentucky, Sept. 17, 1845.

*To the Medical Public.*—The Chair of Obstetrics and the Diseases of Women and Children, in the Medical Department of Transylvania University, is at present vacant; and with a view to fill it in the best possible manner, applications for the appointment are invited from the medical profession. Communications on the subject must be sent to the Dean of the Medical Faculty, prior to the 30th day of January next, when the selection will be made. It is proper to state, that the successful candidate will be required to make Lexington the place of his permanent residence, and that the name of no other applicant will be made public.

M. C. JOHNSON,
Chairman of the Board of Trustees,
Transylvania University.

